# FGF23 in Acute and Chronic Illness

**DOI:** 10.1155/2015/358086

**Published:** 2015-09-28

**Authors:** Christian Schnedl, Astrid Fahrleitner-Pammer, Peter Pietschmann, Karin Amrein

**Affiliations:** ^1^Division of Vascular and Interventional Radiology, Department of Radiology, Medical University of Graz, 8036 Graz, Austria; ^2^Department of Endocrinology and Metabolism, Medical University of Graz, 8036 Graz, Austria; ^3^Department of Pathophysiology and Allergy Research, Center for Pathophysiology, Infectiology and Immunology, Medical University of Vienna, 1090 Wien, Austria

## Abstract

FGF23 is a bone-derived phosphaturic hormone that may become a useful biomarker for the identification of high-risk patients in chronic but also acute disease. It rises early in chronic kidney disease and is strongly and independently associated with excess morbidity and mortality. Emerging data suggest that FGF23 is also elevated in different scenarios of acute illness. In this review, we give an overview on the role of this interesting disease marker and potential and proven interventional strategies and discuss a blueprint for future research.

## 1. Introduction

Fibroblast growth factor 23 (FGF23) has first been described in context with the following orphan diseases: tumor induced osteomalacia, X-linked hypophosphatemia, and autosomal dominant hypophosphatemic rickets (ADHR). In these entities, elevated FGF23 levels are the major etiological factor and cause severe disturbances in bone and mineral metabolism. Based on these observations, it has been established that the osteocyte lacunocanalicular network can function as an endocrine system, targeting distant organs such as the kidney [1].

Research during the last years identified FGF23 as a powerful predictor for clinical outcome, not only in chronic kidney failure but also in a variety of acute and chronic illnesses.

## 2. FGF23 Basics

### 2.1. Pathophysiology of FGF23

Despite the fact that osteocytes are the most abundant type of bone cells, the role of osteocytes as endocrine cells only recently has been discovered. Osteocytes express several molecules that are involved in phosphate metabolism, for example, fibroblast growth factor 23 (FGF23), phosphate-regulating gene with homologies to endopeptidases on the X chromosome (PHEX), matrix extracellular phosphoglycoprotein (MEPE), and dentin matrix protein-1 (DMP-1) [1, 2]. Beside osteocytes, FGF23 is also expressed by osteoprogenitor cells, osteoblasts, cementoblasts, odontoblasts, and chondrocytes [3, 4]. FGF23, in concert with PTH and 1,25(OH)_2_vitamin D, is a major systemic regulator of phosphate homeostasis.

In kidney cells FGF23 binds to a complex of the FGF receptor and Klotho [5]. FGF23 inhibits the sodium-phosphate cotransporters 2a and 2c and thus augments urinary phosphate excretion; an excessive loss of phosphate in the urine results in hypophosphatemia [6, 7]. Moreover, FGF23 decreases mRNA levels of 1alpha-hydroxylase in the kidney [7] and negatively regulates PTH expression in parathyroid cells [8]. Interestingly, both PTH and 1,25(OH)_2_vitamin D were reported to increase the expression of FGF23; thus there are several negative feedback loops between the three major regulators of phosphate metabolism [5].

FGF23 deficient mice exhibit hyperphosphatemia (due to increased renal phosphate reabsorption), hypervitaminosis D (as a result of augmented 1alpha-hydroxylase expression), reduced bone turnover, and an increased osteoid volume [9]. Stubbs et al. reported on severe vascular calcifications and early death in FGF23 deficient mice; a low-phosphate diet improved survival and prevented calcifications [10]. Mice transgenic for human FGF23 exhibit increased phosphate excretion, hypophosphatemia, hyperparathyroidism, and bone mineralization defects [6].

Several diseases observed in humans are due to excessive FGF23 bioactivity. A gain of function mutation of FGF23 leads to autosomal dominant hypophophatemic rickets. Tumor induced osteomalacia (TIO) is an example of an acquired FGF23 related disease. TIO is caused by (mesenchymal) tumors that produce FGF23 and consequently lead to hypophosphatemia and severely impaired bone mineralization [11].

Recently, it was shown that FGF23 is also able to inhibit extrarenal vitamin D activation, namely, 1,25D, and the antimicrobial molecule LL37 synthesis in peripheral blood mononuclear cell monocytes [12]. This could translate to increased susceptibility to infections.

FGF in serum has a half-life of ~20 minutes and this is moderately increased to ~30 minutes in acute kidney injury [13]. Khosravi et al. reported in humans a slightly longer circulating half-life of serum FGF23 (range 46 to 58 minutes) [14].

**Figure 1:**
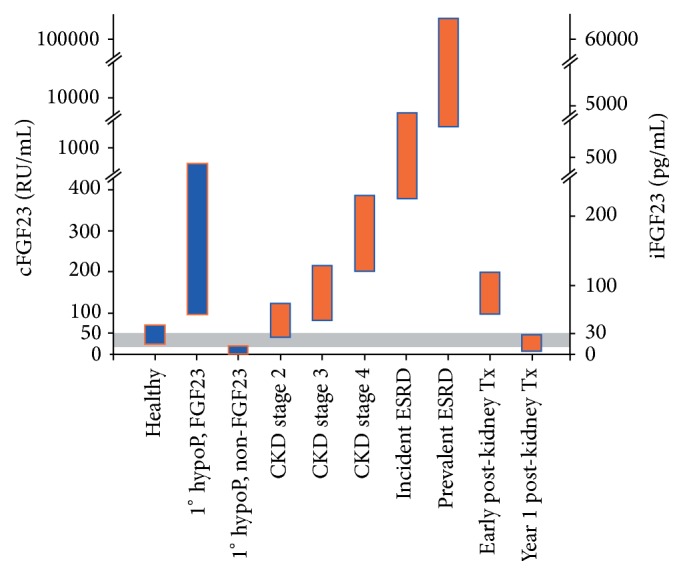
Representative levels of FGF23 in health, in various states of CKD (orange bars), and in primary hypophosphatemic disorders (blue bars). CKD, chronic kidney disease; ESRD, end-stage renal disease; Tx, transplantation. Reproduced with permission from Wolf [15]. The dual *y*-axis presents FGF23 levels on the scales of the two commercially available assay platforms. The intact assay detects biologically intact FGF23 exclusively (iFGF23), whereas the C-terminus (cFGF23) assay is capable of detecting both the intact molecule and its C-terminal fragments. The grey area represents the presumed but incompletely defined normal ranges. “1° hypoP, FGF23” refers to hypophosphatemic disorders caused by primary FGF23 excess, for example, X-linked hypophosphatemia. “1° hypoP, non-FGF23” refers to hypophosphatemic disorders caused by mechanisms other than FGF23 excess, for example, hereditary hypophosphatemic rickets with hypercalciuria, in which FGF23 levels are secondarily suppressed. CKD, chronic kidney disease; ESRD, end-stage renal disease; Tx, transplantation.

Typical ranges for FGF23 serum concentrations in health and illness are given in Figure 1.

### 2.2. Direct Toxicity of FGF23 in Animal Models

In a rodent model, Faul et al. showed that FGF23 seems to be directly toxic independent of Klotho. Isolated rat cardiomyocytes became hypertrophic by FGF23 exposure, wild-type mice developed LVH when exposed to FGF23, and treatment with a FGF23 blocker improved LVH [17].

## 3. FGF23 in Chronic Illness

In several epidemiologic studies dealing with different populations including community-dwelling subjects, patients after kidney transplantation and before dialysis CKD, and even non-CKD patients, FGF23 has consistently been shown to be a strong and independent predictor of mortality [18–23].

In 2008, Gutiérrez and colleagues first reported an exaggerated mortality risk in the large prospective ArMORR cohort (*n* > 10,000) involving end-stage renal disease patients beginning hemodialysis followed up for one year [24]. Independently of phosphate levels, an almost 6-fold higher risk of death was seen in the highest FGF23 quartile (FGF23 levels > 4010 RU/mL) compared to those in the lowest (<1090 RU/mL), and this relationship was linear. An interesting secondary observation in this study was that blacks had significantly lower FGF23 levels. In coronary artery disease, FGF23 has also been implicated with a higher risk for mortality and cardiovascular events [20]. This association remained significant after adjustment for traditional CVD risk factors, C-reactive protein levels, and kidney function.

In line with this, in the LURIC cohort, a large population undergoing coronary artery angiography (mean age of participants 63 ± 10 years), FGF23 levels were independently predictive for all-cause and cardiovascular mortality for a follow-up of 10 years in almost 3000 patients. Median FGF23 serum levels were 54 (40–78) RU/mL. Age- and sex-adjusted hazard ratios (HRs) in the fourth quartile compared to the first quartile of FGF23 were 2.5 for all-cause and for cardiovascular mortality. These associations were robust and independent of other cardiovascular risk factors and serum phosphate [25].

Even in the general population, FGF23 levels are associated with death, cardiovascular disease (CVD), and heart failure (HF). More than 3100 ≥ 65-year-old community-dwelling individuals were followed for over 10 years and 1,730 deaths, 697 incident HF events, and 797 incident CVD events were recorded. High FGF23 was associated with each outcome, but the associations were even stronger in CKD (*n* = 1,128): in this subgroup, compared with the lowest quartile, the highest FGF23 quartile had adjusted hazard ratios (HRs) of 1.9 for all-cause death, 1.9 for incident HF, and 1.5 for incident CVD events, while HRs without CKD were 1.3 for mortality, 1.4 for HF, and 1.1 for CVD [26].

### 3.1. FGF23 in Chronic Kidney Disease

In chronic kidney disease (CKD), elevated FGF23 levels are associated with worse outcomes [27]. FGF23 levels increase early with deteriorating kidney function, even before parathyroid hormone and phosphorus levels rise [28, 29].

In the observational Chronic Renal Insufficiency Cohort (CRIC) study in 3,879 participants with CKD stages 2-3, FGF23 was an independent risk factor for kidney failure and death [18]. In another large observational study in 1,099 HOST (the Homocysteine in Kidney and End-Stage Renal Disease) study patients with CKD stages 4-5, FGF23 was also an independent risk factor for cardiovascular events, dialysis initiation, and all-cause mortality [19]. Accumulating evidence suggests that FGF23 also is a key factor in the development of CKD-MBD [30]. High FGF23 levels have been associated with other complications in this setting such as atrial fibrillation, heart failure, and vascular calcification.

### 3.2. Cardiovascular Disease

The link between elevated FGF23 levels and cardiovascular disease is now well established, particularly in CKD.

#### 3.2.1. Cardiac Surgery

**Figure 2:**
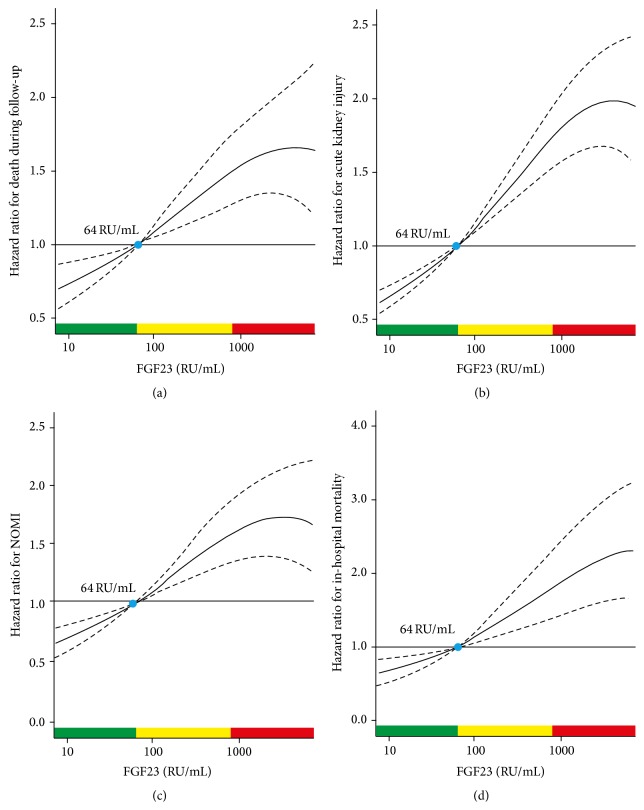
Plot of multivariable adjusted hazard ratios according to preoperative levels of fibroblast growth factor 23: (a) mortality during follow-up, (b) acute kidney injury, (c) nonocclusive mesenteric ischaemia (NOMI), and (d) in-hospital mortality. Solid lines represent the hazard ratios (HRs) and dashed lines the respective 95% confidence intervals. The median of fibroblast growth factor 23 (FGF23) (64 relative units (RU)/mL) was chosen as the reference value (HR = 1). Data are adjusted for age, sex, mean arterial blood pressure, sinus rhythm, coronary artery disease, chronic heart failure, diabetes, serum creatinine, and serum high-sensitivity C-reactive protein. Green areas represent the range of FGF23 with HR < 1, yellow areas with HR ≥ 1 and <1.5, and red areas with HR ≥ 1.5. Reproduced with permission from Speer et al. [16].

Recently, a single preoperative FGF23 level was demonstrated to be an excellent outcome predictor in 859 elective cardiac surgery patients, with a receiver operating curve for mortality greater than the EuroSCORE II, which is a well-established scoring system using 18 variables to predict outcomes in this setting. The area under the curve was 0.80 for FGF23 versus 0.73 for the EuroSCORE II. Moreover, FGF23 was better than NT-proBNP and independently predicted hospital length of stay, duration of mechanical ventilation, and mesenteric ischemia. Although left ventricular ejection fraction was similar between FGF23 tertiles, NT-proBNP levels were sixfold higher in the high versus the low tertile. Patients requiring operative revision and those with endocarditis also had higher FGF23 levels [16]. The relationship between FGF23 levels and different clinical outcomes is depicted in Figure 2.

#### 3.2.2. Cardiogenic Shock and Severe Heart Failure

In a predefined subgroup of the IABP SHOCK II trial (*n* = 182), FGF23 was analyzed on days 1, 2, and 3 in patients with cardiogenic shock caused by acute myocardial infarction.

At all time points, survivors had significantly lower FGF23 levels compared to nonsurvivors, and FGF23 levels above the median (395 RU/mL, IQR 102, 2395) were associated with increased mortality at 30 days and 1 year. Even in a multivariate regression model, FGF23 remained an independent mortality predictor. This was however significant only in patients with poor kidney function (creatinine levels above the median) [31].

In a large cohort of 305 cardiac transplant candidates with end-stage heart failure, FGF23 independently predicted need of mechanical circulatory support, transplantation, or death [32].

Recently, however, the same group reported that, in 154 patients with implanted left ventricular assist devices, 99% had FGF23 values above the reference range (100 RU/mL), but there was no association between stroke or mortality risk and FGF23 levels [33].

### 3.3. FGF23 and Metabolism

FGF23 has been associated with fat mass and dyslipidemia [34], as well as insulin resistance in CKD [35]. Besides, FGF23 levels have been reported to be higher in obesity and decrease with weight loss [36].

## 4. FGF23 in Acute Illness

To date, FGF23 has mainly been studied in chronic disease, particularly in chronic renal failure. There are interesting but limited data on the emerging role in different acute illnesses as outlined below. The short half-life of FGF23 could translate to rapid increases during acute illness and as such FGF23 may become a useful disease marker in acutely and critically ill patients.

*Associations of Elevated FGF23 Levels with Different Endpoints in Different Settings*

*Chronic Illness*

 Mortality.
 Chronic kidney disease.
 Progression to dialysis.
 Left ventricular hypertrophy.
 CKD-MBD.
 End-stage heart failure.

*Acute Illness*

 Mortality.
 Acute kidney injury.
 Nonobstructive mesenteric ischemia after cardiac surgery.
 Sepsis severity.
 Cardiogenic shock/acute myocardial infarction.

### 4.1. Acute Kidney Injury

Similar to CKD, FGF23 serum levels increase rapidly in acute kidney injury (AKI) in animals and humans [13]. In a murine model of folic acid- (FA-) induced AKI, a significant FGF23 rise from baseline was evident already 1 hour after injecting FA, earlier than phosphate levels (2 hours) and plasma neutrophil gelatinase-associated lipocalin (NGAL, 6 hours) [13].

Compared with 8 ICU controls without AKI, FGF23 levels were significantly higher in 12 patients with AKI than in those without (median 1948 RU/mL (IQR), 437–4369) compared to 252 RU/mL (IQR, 65–533) in controls (*p* = 0.01) and also in nonsurvivors versus survivors with median levels of 4446 RU/mL (IQR, 3455–5443) versus 544 RU/mL (IQR, 390–1948; *p* = 0.02). There was however no association between FGF23 levels and the severity of AKI [37].

In another study in 30 AKI subjects, similar findings were reported: FGF23 was significantly higher in AKI than controls (median [interquartile range] = 1471 [224–2534] versus 263 [96–574] RU/mL, *p* = 0.003). FGF23 also correlated negatively with 25-hydroxyvitamin D (*r* = −0.43; *p* = 0.001) and 1,25D (*r* = 0.39; *p* = 0.003) and positively with phosphate (*r* = 0.32; *p* = 0.02) and parathyroid hormone (*r* = 0.37; *p* = 0.005). Baseline FGF23 was also significantly associated with the composite endpoint death/dialysis, even after adjusting for age and serum creatinine (11 events; adjusted odds ratio per 1 SD higher in fibroblast growth factor 23 = 13.7, 95% confidence interval = 1.8–108) [38]. Recently, Speer et al. extended these findings to postoperative AKI in an elective cardiac surgery population [16].

### 4.2. Infection and Sepsis

FGF23 inhibits synthesis of the antimicrobial molecule LL37 in peripheral blood monocytes [12]. It may be hypothesized that through this pathway, FGF23 may be able to substantially modulate the immune response in renal failure. In a small prospective cohort study of 30 hospitalized adults with and 30 without AKI, sepsis severity was positively correlated with FGF23 levels [39].

Larger clinical studies on infection risk in relation to FGF23 are not yet available. However, this hypothesis could partly explain the exaggerated infection risk seen in advanced renal failure, especially dialysis patients [40–42].

### 4.3. Burns

In a small cohort study (*n* = 24), Rousseau et al. assessed the course of FGF23 levels in adult burn patients with a burn surface area (BSA) of >10% receiving standard low doses of cholecalciferol supplementation (200–600 IU daily). Over the first weeks, FGF23 and 1,25 decreased, while the low 25(OH)D levels remained unchanged. Interestingly, a strong positive correlation between FGF23 and CRP levels was detected (*r* = 0.59; *p* = 0.003) [43]. In a pediatric population with severe burns (>40% BSA), Klein et al. performed a secondary analysis of frozen samples from a small RCT (*n* = 17) evaluating the effects of intravenous pamidronate and found that FGF23 was undetectable in most patients. The meaning of this and the discrepancy to the above-mentioned adult cohort remain unclear, although the authors hypothesized that osteocytes may be apoptotic [44].

## 5. Intervention Studies: Can FGF23 or Its Detrimental Effects Be Modified?

Because FGF23 is so strongly associated with poor outcomes in many settings and animal models suggest causality, FGF23 reduction may be an effective treatment target. To date, limited data have analyzed the effect of different interventions (Table 1). Isakova et al. recently summarized the available evidence and found FGF23 reductions of up to 40% by pharmacologic and dietary interventions [45].

Of note, successful kidney transplantation often normalizes the extremely elevated FGF23 levels in advanced CKD to almost normal levels <100–200 RU rapidly [29].

Another interesting development is an anti-FGF23 antibody that has been tested for therapy of hypophosphatemic rickets/osteomalacia, where FGF23 excess was the cause for the disease [46]. Small intervention studies have also reported first promising results for the use in humans [47].

In advanced CKD with severe secondary hyperparathyroidism (*n* = 15), parathyroidectomy with forearm autotransplantation has been shown to reduce FGF23 levels besides phosphate levels [48]. This finding has recently been replicated in 13 hemodialysis patients [49].

In CKD patients (mean GFR 32 mL/min), a small crossover study showed that vegetarian (phosphate poor) diet can decrease FGF23 levels already after one week (*n* = 9) [50]. This is consistent with the findings from studies in healthy participants [51].

Vitamin D and metabolites are also able to modify FGF23 levels although the data are conflicting with regard to the effect of native vitamin D (ergocalciferol, vitamin D2 and cholecalciferol, vitamin D3).

Turner et al. evaluated the effect of loading dose of 300 000 IU vitamin D2 in 45 subjects with a low vitamin D status on FGF23 levels and vitamin D metabolites at baseline, months 1, 2, and 3. They found that FGF23 levels were increased by 50% at month 3 and 1,25 levels quadrupled. There was an inverse correlation of FGF23 and 1,25 levels (*r* = 0.32; *p* = 0.036). The authors concluded that increased 1,25(OH)_2_ vitamin D catabolism mediated by FGF23 may explain the higher risk of fractures and falls seen after high loading doses of vitamin D [52–54]. Similar findings were seen by Burnett-Bowie and colleagues [55].

On the other hand, Uzum et al. demonstrated that, in 18 severely vitamin D deficient women, a loading dose of 150,000 IU vitamin D3 followed by a maintenance dose of 880 IU daily with calcium was able to further significantly reduce low FGF23 levels [56].

**Figure 3:**
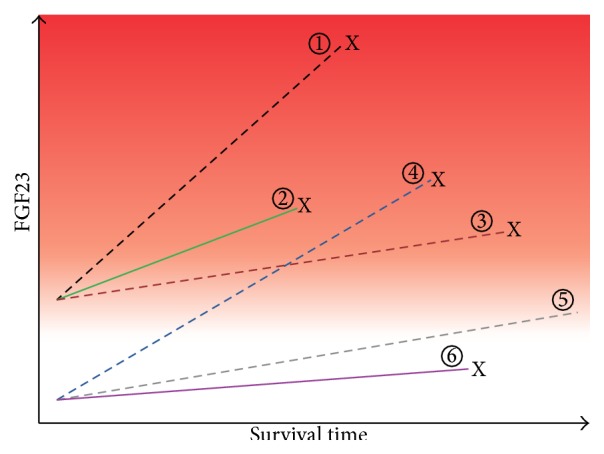
Hypothesis to reconcile the seemingly paradoxical effects of FGF23 and vitamin D on survival in CKD. Reproduced with permission from Wolf [15]. Baseline and change in FGF23 levels are plotted against time among 6 hypothetical patient groups. The spectrum of risk of mortality associated with FGF23 is demonstrated by the red background gradient (higher risk is darker red). Dashed lines represent active vitamin D treated groups, and solid lines represent untreated groups. “X” connotes death. The known effect of elevated baseline FGF23 on risk of mortality is represented by the higher baseline FGF23 and earlier mortality among groups 1–3 versus 4–6. The main effect of active vitamin D therapy on survival is represented by the longer survival of groups 1 and 5 versus 2 and 6. In all groups, FGF23 levels increase with longer duration of ESRD, but the rate of increase is greater among those treated with active vitamin D (greater slopes of FGF23 in groups 1 and 4 versus 2 and 3 and 5 versus 6). The hypothesized interaction between active vitamin D treatment and FGF23 is represented by the significantly greater slopes of increase in FGF23 among active vitamin D treated groups who die sooner compared with those who survive longer (crossing lines of groups 4 versus 3). Thus, it is hypothesized that survival is longest in group 5, which had low baseline FGF23 and received active vitamin D therapy but experienced only a modest increase in FGF23 in response.

Active vitamin D increases FGF23 levels. The seemingly paradox effect of vitamin D analogues on FGF23 and mortality is depicted in Figure 3.

Cinacalcet has been reported to decrease FGF23 levels in hemodialysis patients [57, 58]. Velcalcetide, a novel, long-acting selective calcium sensing receptor agonist, has recently been shown to dose-dependently reduce FGF23 [59].

## 6. Future Research

The role of FGF23 in chronic and more so in acute illness needs to be clarified. As FGF23 seems to be such a powerful and independent predictor for outcomes in CKD and beyond, it may become a useful routine disease marker for the identification of patients with the highest risk for mortality and other complications.

Another fundamental but to date insufficiently answered question is whether FGF23 levels can be modified by any intervention and whether this relates to improved outcomes.

## 7. Conclusion

Fibroblast growth factor 23 is an excellent marker of disease severity and outcomes, particularly in chronic kidney disease. Emerging data suggest a similar role for clinical outcomes in acute illness. Animal models strongly suggest a direct toxic role of FGF. Therefore, FGF23 could be an explanation how bone metabolism affects outcomes in chronic but likely also in acute illness.

Further intervention studies evaluating possibilities to lower FGF23 and their effect on clinical outcomes are urgently needed in CKD, but also other settings.

## 8. Key Points

- FGF23 is a primarily bone-derived phosphaturic hormone that is elevated in acute and chronic illness.
- This relationship has best been established in chronic cardiac and renal disease.
- Relatively higher FGF23 levels are strongly and independently associated with excess morbidity and mortality. However, it is unclear if FGF23 is purely a marker of increased risk or is also contributor.
- Animal models suggest an important pathogenetic role of FGF23 for left ventricular hypertrophy.
- In several studies, FGF23 levels above a threshold of 60–70 RU/mL have been found as a cutoff for increased risk for mortality and other adverse outcomes.
- Limited clinical trial data suggest that FGF23 levels may be modifiable with dietary, pharmacologic, and other interventions.
- Future studies need to clarify the predictive value of FGF23 in different patient populations and evaluate if a reduction of FGF23 serum levels is beneficial for these patients.

## Figures and Tables

**Table 1:** Interventions that may modify FGF23 levels.

Vegetarian diet	Decrease
Phosphate restriction	Decrease
Phosphate binders	Decrease
Vitamin D analogues (see also Figure 3)	Increase
Native vitamin D	Controversial
Parathyroidectomy	Decrease
Cinacalcet	Decrease
Velcalcetide	Decrease
Kidney transplantation	Decrease
Parathyroidectomy	Decrease
